# Whole lung lavage decreases physiological dead space in patients with pulmonary alveolar proteinosis: two case reports 

**DOI:** 10.1186/s13256-023-04085-8

**Published:** 2023-08-19

**Authors:** Tatsuya Fujihara, Atsuko Shono, Nozomi Katayama, Tetsuro Nikai, Yohei Shiratsuki, Yoshihiro Amano, Yoji Saito

**Affiliations:** 1 Division of Emergency and Critical Care Department, Shimane Prefectural Central Hospital, 4-1-1 Himebara, Izumo, Shimane 693-8555 Japan; 2 Department of Intensive Care Medicine, Showa University School of Medicine, Tokyo, Japan; 3 Department of Anesthesiology, Faculty of Medicine, Shimane University, Izumo, Japan; 4 Division of Medical Oncology and Respiratory Medicine Department of Internal Medicine, Faculty of Medicine, Shimane University, Izumo, Japan

**Keywords:** Pulmonary alveolar proteinosis, Whole lung lavage, Carbon dioxide elimination per breath, Enghoff’s dead space

## Abstract

**Background:**

Pulmonary alveolar proteinosis (PAP) is a rare disease characterized by progressive accumulation of the alveolar surfactant. Whole lung lavage (WLL) using a high volume of warmed saline remains the standard therapy. However, no established bedside monitoring tool can evaluate the physiological effect of WLL in the perioperative period. Indirect calorimetry, which is generally used to measure resting energy expenditure, can detect carbon dioxide (CO_2_) production and mixed-expired partial pressure of CO_2_ breath by breath. In this physiological study, we calculated CO_2_ elimination per breath (VTCO_2_,br) and Enghoff’s dead space using indirect calorimetry and measured the extravascular lung water index to reveal the effect of WLL.

**Case presentation:**

We measured VTCO_2_,br, Enghoff’s dead space, and the extravascular lung water and cardiac indices before and after WLL to assess the reduction in shunt by washing out the surfactant. A total of four WLLs were performed in two PAP patients. The first case involved an Asian 62-year-old man who presented with a 3-month history of dyspnea on exertion. The second case involved an Asian 48-year-old woman with no symptoms. VTCO_2_,br increased, and the Enghoff’s dead space decreased at 12 h following WLL. An increase in the extravascular lung water was detected immediately following WLL, leading to a transient increase in Enghoff’s dead space.

**Conclusion:**

WLL can increase efficient alveolar ventilation by washing out the accumulated surfactant. However, the lavage fluid may be absorbed into the lung tissues immediately after WLL and result in an increase in the extravascular lung water.

**Supplementary Information:**

The online version contains supplementary material available at 10.1186/s13256-023-04085-8.

## Background

Pulmonary alveolar proteinosis (PAP) is characterized by the alveolar accumulation of surfactant material and hypoxemia. PAP is caused by the development of antibodies to the granulocyte–macrophage colony-stimulating factor; this inhibits alveolar macrophage activation, thereby causing alveolar surfactant accumulation [1]. Whole lung lavage (WLL) is considered the standard of care for PAP. It can theoretically increase alveolar ventilation and reduce the shunt caused by excess surfactant. Previous studies have examined the improvement of symptoms, chest radiography, oxygenation, vital capacity (VC), and intrapulmonary shunt by scintigraphy to evaluate the effect of WLL [2–4]. However, none of these methods can demonstrate a reduction in the shunt by the washing out of the abnormal surfactant or the increase in the effective ventilation in real-time.

The purpose of this study was to examine the therapeutic effect of WLL on alveolar ventilation, physiological dead space, and extravascular lung water index using indirect calorimetry and pulse index continuous cardiac output (PiCCO) in two patients with PAP.

## Case presentation

### Case 1

**Fig. 1:**
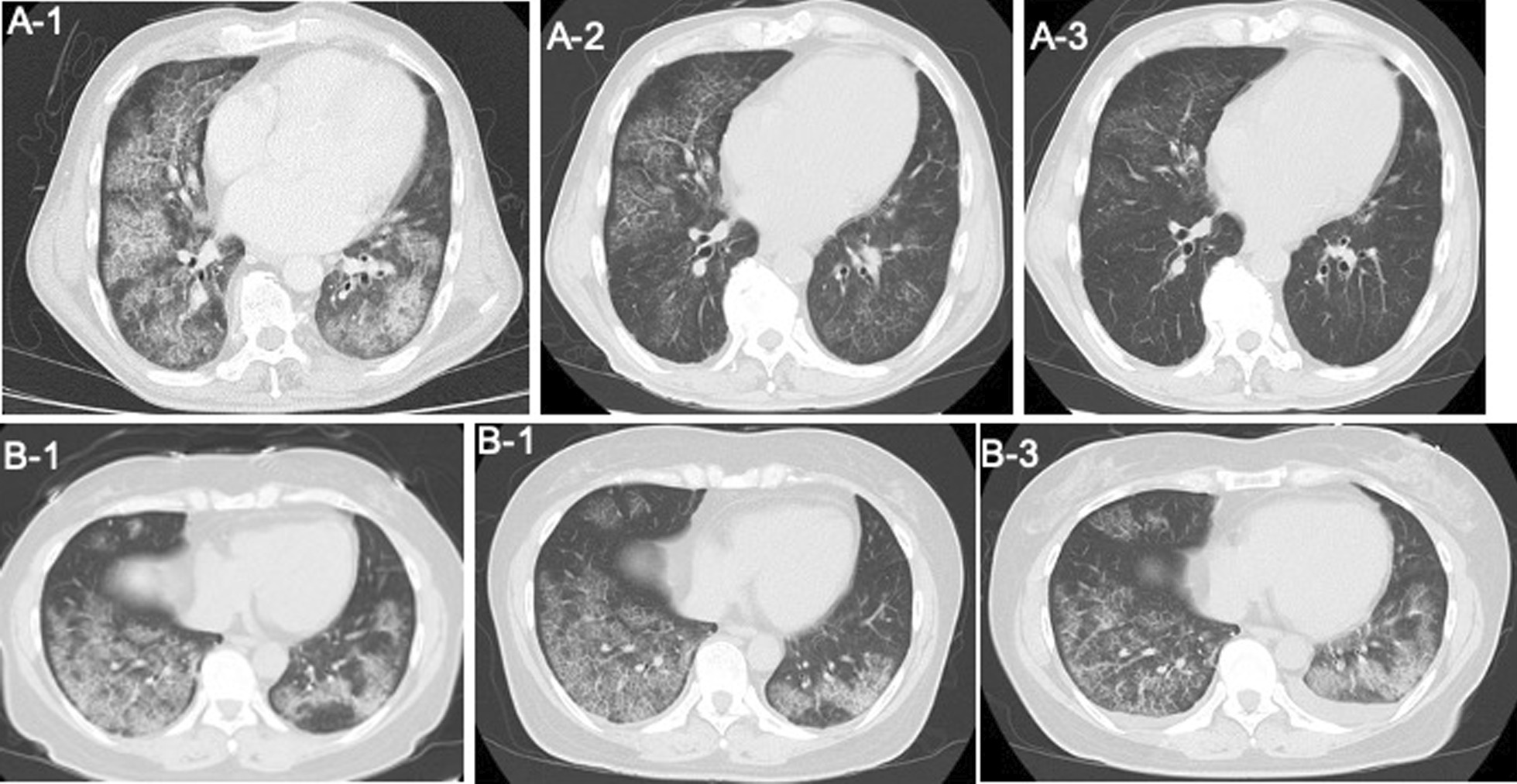
Chest computed tomography and sample collection. Chest computed tomography in **A** Case 1 and **B** Case 2. **A-1**, **B-1** Before WLL: A crazy-paving pattern is observed in both cases. **A-2** At 1 week after left lung lavage. **B-2** At 3 days after right lung lavage. **A-3** At 1 week after right lung lavage. **B-3** At 4 days after left lung lavage

An Asian man in his 60 s presented with a 3-month history of dyspnea on exertion. The patient’s medical history included hypertension and type 2 diabetes mellitus, which were controlled with medication. He was successfully treated for hepatocellular cancer. Computed tomography of the lungs revealed a crazy-paving pattern (Fig. 1A). PAP was diagnosed based on the bronchoalveolar lavage and transbronchial lung biopsy findings. In addition, his GM-CSF antibody was negative. The Hugh-Jones classification was 3, and blood gas analysis (BGA) revealed: mild hypoxemia (partial pressure of arterial oxygen [PaO_2_], 76.8 mmHg; fraction of inspired concentration of oxygen [FiO_2_], 0.21). Pulmonary function testing demonstrated a normal pattern (%Volume Capacity, 83.0%; %forced expiratory volume in 1 s, 103.8%).

### Case 2

The second case was of an Asian woman in her 40 s with no symptoms. An infiltrative shadow was observed on chest radiography obtained 3 years prior (Fig. 1B). The patient was referred to our hospital as the abnormal shadow did not improve, and the diagnosis of PAP was made based on bronchoalveolar lavage findings and genetic testing. She had no GM-CSF antibodies and serum GM-CSF levels of 10.8 pg/mL. Moreover, no phosphorylation of STAT5 was detected under GM-CSF stimulation, suggesting GM-CSF receptor α-chain abnormality. The results of the single nucleotide polymorphism array showed that the region containing the GM-CSF receptor α-chain gene (CSF2RA) was homozygously deleted, and a diagnosis of hereditary PAP due toα-chain dysfunction was made. Although the Hugh-Jones classification was 1, BGA showed mild hypoxemia (PaO_2_, 74.7 mmHg; FiO_2_, 0.21). Pulmonary function testing demonstrated an impaired carbon monoxide diffusion capacity (81% of the predicted value).

#### Anesthesia

WLL was performed under general anesthesia. Standard monitoring was established upon the arrival of the patient to the operating room. General anesthesia was induced with 1.5 mg/kg propofol and 5 mcg/kg fentanyl, and 0.8 mg/kg rocuronium was administered to achieve muscle relaxation. A left-sided double-lumen endotracheal tube was subsequently inserted, and its position was confirmed using a flexible bronchoscope. A leak test was performed at 40 cmH_2_O to prevent lavage fluid leakage into the ventilated lung. An arterial catheter was cannulated into the brachial artery to measure the PiCCO (PV2014L08-A, FUKUDA COLIN Co., Ltd., Saitama, Japan), and a central venous catheter was inserted into the internal jugular vein. Anesthesia was maintained with a continuous infusion of propofol, remifentanil, and fentanyl to sustain the bispectral index values between 40 and 60. One-lung ventilation was commenced before the start of WLL and maintained during the procedure. Patients were continuously ventilated with the volume control mode and a fixed tidal volume of 6–8 mL/kg during and after WLL. The endotracheal tube was replaced with a single-lumen tube after WLL, and the patients were transferred to the intensive care unit.

#### WLL protocol

**Fig. 2:**
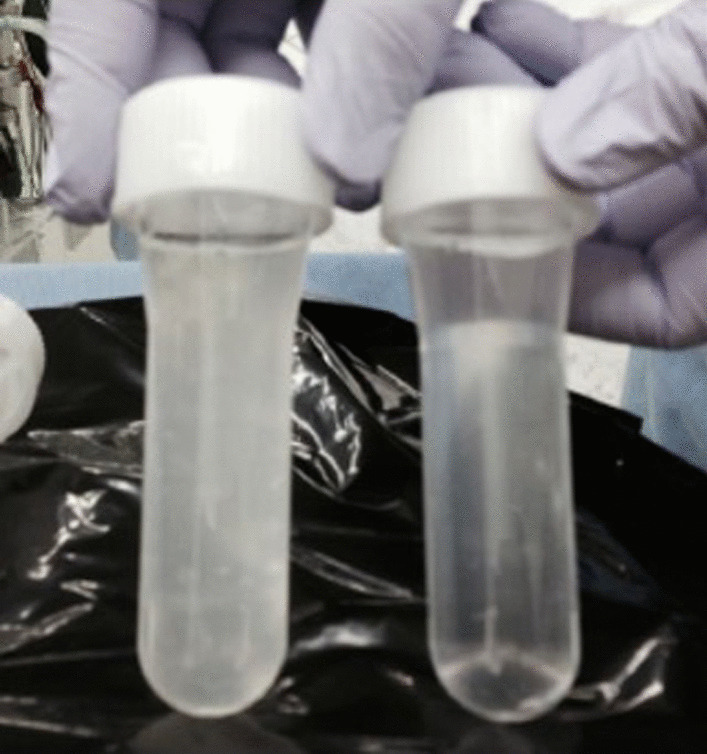
The collected fluid with visible turbidity. The first *(left)* and the last *(right)* saline samples collected are presented for Case 1 (Trial 1). Saline samples collected at earlier time points were whitish because of the presence of surfactant; subsequent samples were progressively clearer

A single trial of WLL was scheduled separately for the right and left lungs in each patient. Under one-lung ventilation, we infused lavage fluid (warmed saline, 37 °C) into the unventilated side of the lung until the infusion reached its capacity; subsequently, the lavage fluid was collected by gravity. The collected turbid fluid contained excess surfactant and proteins (Fig. 2). We performed this procedure repeatedly until the collected fluid was clear.

#### Measurements

Measurements were obtained in the supine position with bilateral ventilation at three time points: before the initiation of WLL (T1), immediately after WLL (T2), and 12 h after WLL (T3). We measured the VTCO_2_,br via indirect calorimetry (Aeromonitor AE-310S, Nihon Kohden Co, Tokyo, Japan), and the dead space was calculated using Enghoff’s modification of Bohr’s dead space formula (VDEnghoff/ VT) as follows:

VDEnghoff/ VT = (arterial CO_2_ tension [PaCO_2_]–Mixed-expired partial pressure of CO_2_ [PECO_2_])/PaCO_2_. [5]

Enghoff's dead space differs from the Bohr’s dead space formula in that it includes the shunt area for the dead space in the calculation in addition to the actual dead space [5]. The extravascular lung water index (EVLWI) and cardiac index (CI) were measured with the PiCCO at each time point. The PaO_2_/ FiO_2_ ratio was also calculated as an indicator for oxygenation. In addition, each operation time and net positive balance of lavage fluid (difference between infused and drained fluid volume) were recorded.

## Result

**Fig. 3:**
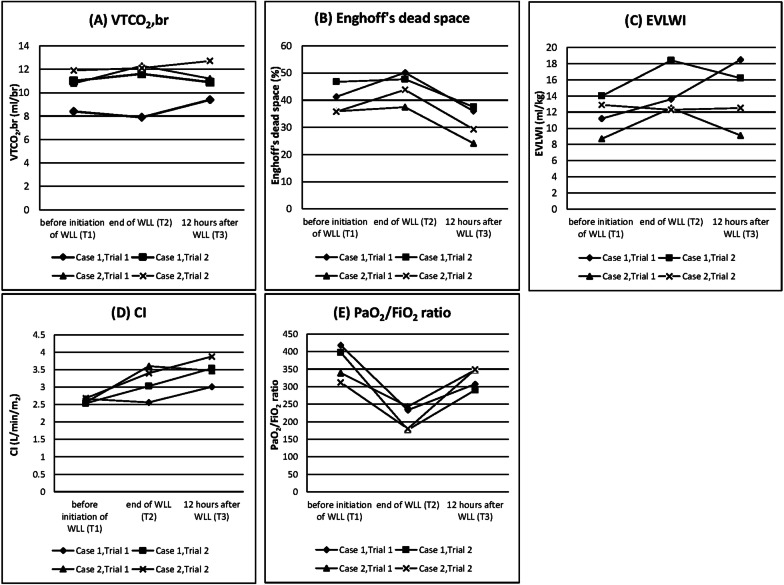
Changes in VTCO_2_,br, Enghoff’s dead space, EVLWI, CI, and the PaO_2_/FiO_2_ ratio in each trial. **A** VTCO_2_,br, **B** Enghoff’s dead space, **C** EVLWI, **D** CI, **E** PaO_2_/FiO_2_ ratio. T2 was measured at WLL completion after confirming the stability of vital signs before ICU admission. T3 was measured 12 h after ICU admission. After WLL, the patients were transferred to the ICU, where they were ventilated and deeply sedated with continuous infusion of propofol, dexmedetomidine, and fentanyl until T3 measurement. Noradrenaline was administered at a rate of 0.03 mcg/kg/min at T2 and T3 measurements only in Trial 2 of Case 1. In all trials, extubation was performed 18 h after WLL. *VTCO*_*2*_*,br* CO_2_ elimination per breath, *EVLWI* extravascular lung water index, *CI* cardiac index, *PaO*_*2*_*/FiO*_*2*_* ratio* partial pressure of arterial oxygen/fraction of inspired concentration of oxygen, *T2* time point 2, *WLL* whole lung lavage, *ICU* intensive care unit, *T3* time point 3

The two patients underwent a total of four trials. The second trial was performed 5 weeks after the first trial in each case. The results obtained for VTCO_2_,br, Enghoff's dead space, EVLWI, and PaO_2_/ FiO_2_ are presented in Fig. 3. The mean VTCO_2_,br at T2 and T3 increased by 3.7% and 5.3%, respectively, compared with that at T1. Enghoff's dead space increased by 12.6% at T2; however, it decreased by 20.8% at T3 compared with that at T1. Mean EVLWI increased from 11.2 mL/kg at T1 to 14.2 mL/kg at T2, and in three trials, it returned to the baseline levels 12 h after WLL (except in one trial). The mean CI increased from 2.6 L/min/m^2^ at T1 to 3.1 L/min/m^2^ at T2 and 3.5 L/min/m^2^ at T3. The remaining parameters for calculating Enghoff's dead space and VTCO_2_,br are presented in Additional file 1: Table S1. While the mean PaO_2_/ FiO_2_ decreased from 367 at T1 to 208 at T2, it recovered to 323 at T3. The operation time, the number of washes for each trial, and the average net positive balance are presented in Table 1. The mechanical ventilator settings are presented in Table 2.

**Table 1:** Wash-in wash-out procedure, infused and uncollected fluid volume, and operation time in each trial

		Lung side	Wash-in, wash-out procedure	Volume of saline	Net positive balance	Time of WLL
Case 1	Trial 1	left	11 times	6000 mL	890 mL	211 min
	Trial 2	right	10 times	10,600 mL	410 mL	357 min
Case 2	Trial 1	right	10 times	3460 mL	1430 mL	239 min
	Trial 2	left	10 times	11,150 mL	690 mL	379 min

WLL: Whole lung lavage

**Table 2:** Ventilator settings in each trial. Patients were constantly ventilated with the volume control mode

Case 1, trial 1	T1	T2	T3
Mode	VCV	VCV	VCV
FiO_2_	0.4	0.6	0.4
Tidal Volume (ml)	375	380	380
PEEP (cmH_2_O)	4	7	7
RR	15	15	15

Case 1, trial 2	T1	T2	T3
Mode	VCV	VCV	VCV
FiO_2_	0.4	0.5	0.45
Tidal Volume (ml)	500	500	500
PEEP (cmH_2_O)	4	4	4
RR	10	10	12

Case 2, trial 1	T1	T2	T3
Mode	VCV	VCV	VCV
FiO_2_	0.4	1.0	0.3
Tidal Volume (ml)	400	400	400
PEEP (cmH_2_O)	4	4	5
RR	10	10	12

Case 2, trial 2	T1	T2	T3
Mode	VCV	VCV	VCV
FiO_2_	0.4	0.6	0.3
Tidal Volume (ml)	450	450	450
PEEP (cmH_2_O)	4	4	5
RR	10	10	10

FiO_2_: Fraction of inspired concentration of oxygen, PEEP: Positive end expiratory pressure, RR: Respiratory rate

## Discussion and conclusions

We performed a total of four WLLs in two patients and evaluated the physiological effect, in which Enghoff’s dead space decreased and the VTCO_2_,br increased slightly 12 h following the completion of WLL.

Previous studies have evaluated the effect of WLL by monitoring the symptoms, chest radiography, oxygenation, VC, intrapulmonary shunt by scintigraphy, and protein concentration in the recovered fluid [2–4, 6, 7]. In addition, a recent report demonstrated that lung ultrasound could be used for bedside assessment of WLL [8]. Furthermore, there have been reports evaluating the ventilation-perfusion ratio using electro impedance tomography (EIT) as a bedside monitoring tool. However, the method for detection of lung perfusion using hypertonic saline and the calculation to determine the degree of physiological dead space and shunt is not well established [9, 10]. Therefore, none of these methods can directly measure the increase in efficient ventilation by monitoring the changes in VTCO_2_,br and Enghoff’s dead space before and after WLL in real-time. PAP causes hypoxia due to an intrapulmonary shunt, which arises because of the accumulation of alveolar surfactant [1]. Therefore, WLL efficacy can be evaluated by a decrease in shunt and an increase in efficient alveolar ventilation. In the present two cases, we used Enghoff’s dead space to assess the reduction in the shunt area. The formula for Enghoff’s dead space is a modification of the Bohr’s dead space equation (VDBohr/VT = (alveolar CO_2_ tension [PACO_2_]-PECO_2_)/PACO_2_). In the presence of a shunt, there is a dissociation of CO_2_ tension between PACO_2_ and PaCO_2_. By replacing PACO_2_ with PaCO_2_, the calculation of Enghoff’s dead space includes the actual dead space (ventilation-perfusion ratio [V/Q] ≒∞) and the area related to the shunt (V/Q≒0). Thus, an increase in Enghoff’s dead space reflects gas exchange impairment because of the shunt and an increase in dead space [5]. This can be used as a physiological parameter to assess the changes in the shunt areas caused by therapeutic interventions. For instance, reductions in Enghoff’s dead space have been used to optimize ventilatory settings in the acute respiratory distress syndrome model or anesthetized patients [11, 12]. Therefore, the therapeutic efficacy of WLL can be assessed by monitoring the reduction of Enghoff’s dead space owing to the removal of excessive alveolar surfactant; this would correspond with an increase in efficient alveolar ventilation.

In the present two cases, Enghoff’s dead space decreased by more than 20%, indicating that WLL removed a sufficient amount of excess alveolar surfactant. Enghoff’s dead space temporarily increased immediately after WLL in some trials, and the EVLWI also increased. As the lavaged fluid was not collected fully, we speculated that fluid remained in the alveolar space immediately after WLL completion and was subsequently absorbed into the pulmonary interstitium over time; this contributed to the transient increase in Enghoff’s dead space and EVLWI at 12 h after WLL. Similarly, the PaO_2_/FiO_2_ ratio did not improve immediately after WLL, and in some trials, it did not even recover to baseline levels after 12 h. Gas exchange impairment may have been attributed to compromised diffusion, as a result of the accumulated lavage fluid in the alveoli and pulmonary interstitium.

Previous studies have reported that VTCO_2_,br is affected by both ventilation and pulmonary blood flow (PBF). Under constant ventilatory conditions, the change in VTCO_2_,br may depend on PBF, that is, the cardiac output [5]. In our study, VTCO_2_,br showed a gradual increase during the study period, and concomitant increases in CI were also detected in all trials. This may indicate that the measurement of VTCO_2_,br was affected by cardiac output to some extent. In addition, the level of inspiratory airway pressures could affect the measurements of dead space and gas exchange by changing PBF and lung mechanics. Therefore, hemodynamic parameters and ventilatory settings should be considered when evaluating efficient ventilation before and after WLL. Furthermore, it should be taken into account that overinflation caused by inappropriate ventilator settings can also influence on the calculation of the dead space.

Thus, WLL could decrease Enghoff’s dead space by washing out the accumulated surfactant in this study. However, the lavage fluid may be absorbed into the lung tissues immediately following WLL and result in an increase in extravascular lung water and impairment of oxygenation.

### Supplementary Information

**Additional file 1: Table S1**. CO_2_ data for the calculation of Enghoff's dead space and VTCO_2_,br at each timepoint.

## Data Availability

The data that support the findings of this study are available from the corresponding author, Tatsuya Fujihara, upon reasonable request.

## Abbreviations

PAP: Pulmonary alveolar proteinosis
WLL: Whole lung lavage
CO_2_: Carbon dioxide
VTCO_2_,br: CO_2_ elimination per breath
PiCCO: Pulse index continuous cardiac output
BAG: Blood gas analysis
PAO_2_: Partial pressure of arterial oxygen
FiO_2_: Fraction of inspired concentration of oxygen
PECO_2_: Mixed-expired partial pressure of CO_2_
PaCO_2_: Arterial CO2 tension
EVLWI: Extravascular lung water index
CI: Cardiac index
T1: Before the initiation of WLL
T2: Immediately after WLL
T3: 12 H after WLL
PACO_2_: Alveolar CO_2_ tension
V/Q: Ventilation-perfusion ratio
PBF: Pulmonary blood flow
