# Supplementary material for: Whole lung lavage decreases physiological dead space in patients with pulmonary alveolar proteinosis: two case reports
Source: J Med Case Rep. 2023 Aug 19;17:353. doi: 10.1186/s13256-023-04085-8 (PMC10439532; doi:10.1186/s13256-023-04085-8)
Supplement: Supplementary file 1 — Additional file 1: Table S1. CO2 data for the calculation of Enghoff's dead space and VTCO2,br at each timepoint. [file 13256_2023_4085_MOESM1_ESM.docx]

*Additional file 1: Table S1: CO_2_ data for the calculation of Enghoff's dead space and VTCO_2_,br at each timepoint.*

| *Case 1 trial 1* | *T1* | *T2* | *T3* |
| --- | --- | --- | --- |
| *VCO_2_ (ml/min)* | *126* | *118* | *141* |
| *VTCO_2_,br (ml)* | *8.4* | *7.9* | *9.4* |
| *PaCO_2_ (mmHg)* | *42.7* | *43.1* | *46.3* |
| *PECO_2_ (mmHg)* | *25.1* | *21.5* | *29.6* |
| *Enghoff's dead space (%)* | *41.2* | *50.1* | *36* |

| *Case 1 trial 2* | *T1* | *T2* | *T3* |
| --- | --- | --- | --- |
| *VCO_2_ (ml/min)* | *88* | *116* | *131* |
| *VTCO_2_,br (ml)* | *11* | *11.6* | *10.9* |
| *PaCO_2_ (mmHg)* | *36* | *47.2* | *43.5* |
| *PECO_2_ (mmHg)* | *19.2* | *24.7* | *27.2* |
| *Enghoff's dead space (%)* | *46.7* | *47.7* | *37.5* |

| *Case 2 trial 1* | *T1* | *T2* | *T3* |
| --- | --- | --- | --- |
| *VCO_2_ (ml/min)* | *108* | *123* | *134* |
| *VTCO_2_,br (ml)* | *10.8* | *12.3* | *11.2* |
| *PaCO_2_ (mmHg)* | *42.9* | *50.8* | *44.8* |
| *PECO_2_ (mmHg)* | *27.5* | *31.8* | *34* |
| *Enghoff's dead space (%)* | *35.9* | *37.4* | *24.1* |

| *Case 2 trial 2* | *T1* | *T2* | *T3* |
| --- | --- | --- | --- |
| *VCO_2_ (ml/min)* | *119* | *121* | *127* |
| *VTCO_2_,br (ml)* | *11.9* | *12.1* | *12.7* |
| *PaCO_2_ (mmHg)* | *41.7* | *42.9* | *49.1* |
| *PECO_2_ (mmHg)* | *26.8* | *24.1* | *34.7* |
| *Enghoff's dead space (%)* | *35.7* | *43.8* | *29.3* |

The measurement by indirect calorimetry for PECO_2_ and VTCO_2_,br was performed for 5 mins at each time point. PECO_2_ measured in a single breath during stable respiratory cycles with a constant expired tidal volume was applied for the calculation of Enghoff's dead space. VTCO_2_,br was calculated by dividing the minute elimination of CO_2_ (ml/min) measured by indirect calorimeter by respiratory rate.

Abbreviations: VTCO_2_,br, CO_2_ elimination per breath; PECO_2,_ mixed-expired partial pressure of CO_2._
